# American Dental Association

**Published:** 1890-11

**Authors:** 


### Reports of Society Meetings.

### AMERICAN DENTAL ASSOCIATION.—THIRTIETH ANNUAL MEETING, EXCELSIOR SPRINGS, MO., AUGUST 5 TO 8, 1890.

(Continued from page 617.)

*Second Day's Proceedings.*

The second day's session of the American Dental Association convened at the opera-house at Excelsior Springs, Mo., at 10 A.M., Wednesday, August 6, First Vice-President A. W. Harlan, of Chicago, presiding.

The committee on the S. S. White matter reported that the communication from the company concerning the publication of the reports was substantially correct. The report was adopted.

The committee on voluntary essays reported that the various matters in the president's address had been referred to the different subdivisions. The report was adopted.

Dr. C. N. Peirce, chairman of the Section on Dental Education, Literature, and Nomenclature, then made his report. He stated at the commencement, that there had been a steady growth in the interest taken in matters of education. The number of dental colleges at the time of the last annual report was thirty-one. This number was decreased during the year by the suspension of the dental department of the St. Louis College of Physicians and Surgeons, but increased by the establishment of the dental department of Tennessee Medical College, at Knoxville, Tenn., the Western Dental College, at Kansas City, Mo., and the United States Dental College, at Chicago. The number of dental colleges in active operation at the present time being thirty-three.

At the commencements held[1] since the last meeting of this association, from twenty-nine of these colleges there were graduated nine hundred and sixty-three persons, an increase of one hundred

[1] No report was received from the Northwestern College of Dental Surgery and the National University (2), while the Western Dental College and the Dental Department of the Tennessee Medical College have held no commencement (2). Total 4.

and sixty-seven over the preceding year. The ratio of increase in the number of graduates this year is three times as great as it was last year over the year preceding it.

The total number of graduates during the past years (1886–1890 inclusive) is three thousand six hundred and five.

The rapid multiplication of dental schools in the United States involves a question which is menacing the future prosperity of the dental profession. The results in all probability will be similar to the results attained by the medical schools of this country who turn out annually, to prey on an unsuspecting public, a number of men which largely exceeds the number of graduates in Great Britain, France, Germany, Italy, and Austria combined. This has been carried to such an extent that the medical graduate is the laughing-stock of his transatlantic *confrère.* He now swarms in such numbers in the United States that one out of every six hundred is a medical graduate. At the present rate of progress made by the dental schools of the United States, they will soon equal, in fertility at least, the medical colleges of this country.

While eventually the subject of education will probably be under national control, at the present time some means ought to be adopted whereby the rapid increase of so-called colleges might be checked. Possibly the only remedy at present lies in the amendment of State constitutions, so that at least none but "reputable and capable" men may be able to secure articles of incorporation. In one State the incorporation of dental colleges and infirmaries is almost coequal with the issuance of dog licenses. The section is not prepared to offer any special remedy, but hopes that members of the Association may reflect on the question and be prepared to adopt some means to check the evil before its serious consequences become more marked. To one subject the section would direct attention. There is to-day, in the literary and scientific elements of society, an effort being made to establish a national university at Washington, D. C. As that idea matures, which will probably be in the not-far-distant future, those interested in the progress of dentistry should be on the alert and see that our profession is there represented.

The advantages offered for the establishment of such an institution in the capital of our country are too patent to be overlooked. First, the climate for eight months in the year is hardly surpassed in our borders. It is the home of men of talent,—students of profound and varied ability and of institutions such as the Smithsonian National Museum, Government Surveys and Commissioners,

American Medical Museum, philosophical societies, national geographical, agricultural, and chemical libraries, embracing over one million volumes, and laboratories embracing illustrative material in every department of medicine and surgery.

With anxiety and interest every earnest member of the dental profession is looking to the action of the National Association of Dental College Faculties. The adoption last year by that body of a resolution requiring all schools connected therewith to adopt a three-years' graded course of not less than five months for the session of 1891 and 1892, to be continued thereafter by all such schools, was certainly a forward step, which, it is to be hoped, will be fruitful of much good. That this step shall be maintained, so that the profession may reap the benefit of this united and progressive action, depends largely upon the State Boards of Dental Examiners and the encouragement they receive from the National Board of Examiners. It is an easy matter for schools to obtain representatives in the Association of Dental College Faculties, and advertise in their announcement that their requirements are consistent and in accord with said association, and subsequently in the admission and graduation of students to ignore every vital principle therein involved. The State Examining Boards are the only power capable of correcting this malefeasance, and unless they are willing to exercise this power the resolutions proclaiming this advance in educational institutions are but blots upon their books, and as worthless as a discarded garment. It is well known that a prominent school has so advertised its accord with the Association of Faculties and at the same time has violated the latter's requirements when a few students were to be gained thereby, and yet their diplomas have been endorsed as freely as those from schools where every letter has been conscientiously adhered to.

Since the last meeting of this body three States have so modified their dental law as to require an examination and endorsement of all who propose to practise within their borders or commonwealth without regard to where the graduate's diploma or certificate was acquired. Mississippi, New Jersey, and Minnesota are the States which have recently taken this step, Massachusetts having three years previously inaugurated this advance which your chairman of the section believes to be in the right direction. State Board of Examiners share with the faculties an important responsibility, and such action on their part must prove a great incentive to students to be thorough and well-grounded in principles and practice. Of course, the value of this State Board examination, as well as its justice, depends upon the qualifications of those conducting it. It is not enough that the members of this Board of Examiners enjoy the emoluments of a large and remunerative practice, or that they be held in good repute by their neighbors, and have the degree of D.D.S. previously obtained from some college. Any one or indeed all of these are not necessarily qualifications for the proper performance of such a function. The qualifications of the State *critic* or *examiner* must depend upon judgment. This judgment must be ripened or trained through what may be termed first-hand knowledge, knowledge the result of practical experience and experimentation.

One with a very limited experience may judge of the density and finish of a filling or a piece of prosthetic dentistry, but may be very poorly qualified to criticise the appropriateness of the one or adaptation of the other. An eminent professor has said, "Our knowledge of the universe depends upon our contact with it, and may be expressed in what we call science." So, one's knowledge of dentistry depends not only on his familiarity with the materials used, but also on the knowledge possessed of the tissues or organs upon which the operations are performed. So, just in proportion to the extent of this knowledge gained from contact is the knowledge of the science of dentistry broad or narrow.

If it were only practical skill, or skill in technique, that is required, the student need not go beyond the preceptor's office and laboratory; but fortunately our colleges are instituted for a deeper and broader purpose. The forces of nature, as embraced in biology, chemistry, metallurgy, etc., should be and are taught in our best schools, as much as a matter of necessity in the lecture-room, clinics, and laboratory, as the more simple mechanical processes. The pertinent question now arises: How many of our average practitioners, whose graduation dates back *five*, *ten*, *fifteen*, or *twenty years*, of whom our examining boards are composed, are qualified to judge of more than the superficial qualifications of the student; and how often is injustice done the applicant through their ignorance and inability to open the chambers or recesses of valuable information? The advantage which is to be gained in the prospective three-years' graded course, soon to be adopted by our schools, is the opportunity for additional scientific training which it offers.

Some four years ago, when your chairman of Section Two had the honor to hold the same position, he prepared a paper looking towards a higher standard than the degree of D.D.S. now conferred by the colleges. It was at that time deemed by the section premature or utopian; but at the present time the same ideas are accepted by the section as worthy of voice, and are as follows:

In view of the fact that a very large proportion of our dental schools are dependent upon the class in attendance for support, and that the compensation to professors and demonstrators has a like sustenance, the temptation is constantly to increase the class beyond capacity for instruction, and to estimate its value by numbers rather than quality. To such an extent has this been true, in the desire to excel by publishing a large graduating class, that a prominent school some two or three years since graduated the same five students two consecutive years. To express the condition of our educational institutions tersely and in a few words, we should say that, under the present organization of a very large majority of our schools, the tendency or temptation is to insufficiency rather than proficiency, to superficiality rather than solidity. Therefore we deem it an imperative necessity for growth and proficiency that there should be established a higher standard than is now required by any of our dental or medico-dental schools, and that this association now in session should before adjournment appoint a committee of five to consider the advisability of establishing a national board, who would be clothed with power to confer a title or degree upon those members of our profession who had attained a well-rounded degree of proficiency by experimental and true scientific work to make them worthy of such distinction. The object or purpose of this action would be towards a broader, a more liberal education in everything that pertains to the profession of dentistry.

At the last annual meeting the following resolution was adopted:

*Resolved*, That Section II. be instructed to formulate and present at the next meeting of this Association a plan of work for the sections of the Association.

Many of the members of this Association are not satisfied with the method now in vogue of doing the work of the Association, and the resolution was adopted, in all probability, for the purpose of having some means devised whereby the annual labors of the Association may be made more valuable.

An attempt was made by the secretary to secure reports of the meetings of the various State and local societies, to get abstracts of the papers read before them and of the discussions following. The secretary issued the following circular-letter to secretaries of societies:

To Secretaries of Dental Societies in the United States:

A plan, under which the work of various sections of the American Dental Association should be done, is being elaborated by the section to which this subject was referred at the last meeting of the Association. The secretaries of the various dental societies are requested to prepare (or have some competent member of the society do so) a brief, concise synopsis of the work of the society. This should include extracts from the papers read and from the discussions following, and should include mention of every item of scientific value brought forward during the meeting of the respective societies, a description of new instruments, appliances, and in fact a mention of *everything* worthy of notice. These reports should be sent as soon as possible after the adjournment of the meeting of the respective societies to the secretary of Section II. A. D. A. (Louis Ottofy, 70 Dearborn Street, Chicago). They will then be separated and sent to the respective chairmen of the various sections. In this way the chairman of each section will receive a report of everything that has been accomplished by the different societies of the United States in the particular branch represented by his section. The sections will then prepare their reports from these State and local reports, and thus present an entire exhibit of the profession's advance and labor during the year. The societies which contribute to the accomplishment of this object will receive due credit in the report of each section.

Please forward at once to the undersigned any papers or essays, or abstracts of them, read during the past year, for the purpose herein mentioned.

Credit will be given your organization for the work done during the year.

Address all communications to Louis Ottofy,

*Secretary Section II. A. D. A.*, 70 Dearborn Street, Chicago.

The intention was to secure the scientific reports to distribute to the respective sections, in order that they might present whatever they found worthy in their report to this Association. But few replies were received. This led to an inquiry regarding the American Dental Association, and the relation which it bears to the dental societies, and the prefession generally.

The investigation disclosed the following facts:

1. That the American Dental Association is not a truly representative body of the profession.

2. That its membership is too small to accomplish what it should.

3. That every dental society ought to be represented by one or more delegates, and that these delegates should bring to it a report of the entire work of their societies during the preceding year, and thus cause the reports of these sections to contain a perfect epitome of the entire scientific doings of the profession in the United States.

In securing a list of members of all dental societies in the United States, we find that there are not less than ninety, nor more than one hundred, dental societies—strictly speaking and excluding alumni and other kindred organizations—in the United States. Every State, except Montana, Nevada, Oregon, and Washington, has a State society, and nearly all of them one or more local societies. The District of Columbia has two dental societies. Neither of the eight Territories has any dental society.

The approximate aggregate of membership in these ninety odd societies is between three thousand and three thousand five hundred. These societies are classed as national, semi-national, interstate, State, local, county, and city societies. This certainly large membership was represented last year in this Association by only thirty-eight delegates, who represented twenty-two societies which are located in twelve States and the District of Columbia. The dentists of eighteen States and eight Territories were not even represented in the membership of the Association by any one. Three States contribute seventy-five members, while the remaining twenty-three States contribute only one hundred members. It will also be noted that, with the exception of twenty-three who may be credited to the West, two to the Northwest, and fourteen who are from the South, the Association is made up of one hundred and thirty-six dentists from the Eastern States, making it appear more local than national.

This condition ought not to be tolerated. The strength and power of the Association must come from unity. Some means should be adopted whereby the membership of the Association may be increased to at least one thousand. An invitation or an appeal ought to be issued to every society to send delegates to the meetings of this Association, even if it shall be necessary for the local societies to pay fair compensation to their representative. When this can be accomplished, the section would recommend the following method of work to the sections:

Each section should appoint some one of its members, or delegate some one, to attend the meetings of dental societies and make reports of their proceedings, report important clinics, note new appliances and methods, and make a report to their respective sections, or make one entire report, to be divided, and each section to be given those parts relating to its own special work. In addition to these reports, abstracts of papers published in various journals should be made. The sections then ought to sift out the chaff, and present to the Association a summary of everything that has occurred affecting dental science in any way, direct or collateral.

The length of this report makes it impossible to present much in relation to dental literature. Among the most prominent of works published during the year is a treatise on the irregularities of the teeth and their correction, designed for practitioners and students, by J. N. Farrar, M.D., D.D.S., of New York City. This publication, when complete, will be one of the most remarkable and creditable productions which the present decade has witnessed. It will consist of three volumes,—divided for the convenience of the student into several parts.

The first volume deals with the history and etiology of the subject; the basal principles of regulation; nomenclature; principles of construction of apparatus; retaining devices; laboratory rules for manufacturing devices; application of force; eruption; antagonism; interdental spaces; and the correction of irregularities by grinding and by extraction.

The second volume contains the classification of irregularities and the various methods of their treatment, such as straightening teeth to line, turning and elevating teeth, widening and enlarging the dental arch, and the correction of protruding teeth.

The third volume is largely pictorial, being an object index of all mechanisms described in the other volumes. While in Volume II. the order is governed by the locality and kind of operations, in Volume III. classification is made according to the principles upon which each mechanism is constructed. Those acting by *springs*, or *gradual*, in one class, and those by *screws*, or *positive*, in the other.

The author of this publication has been at an expense of nearly seven thousand dollars ($7000) in the production of upward of two thousand (2000) engravings.

The first volume will be complete for the purchaser by October 1; the second and third will soon follow, as the material is now all in the hands of the publisher.

There is nothing to report on dental nomenclature save a paper noted from Dr. W. H. Atkinson. The section recognizes that little can be accomplished upon this subject save it is considered by some international organization.

The section presents for your consideration the following papers: "Dental Education, Nomenclature, and Terminology," by W. H. Atkinson, of New York City; "Scientific Instruction in our Colleges," by A. H. Thompson, of Topeka, Kansas; "Dental Education," by C. B. Atkinson, of New York City; "National Dental Education," by B. A. R. Ottolengui, of New York City.

(To be continued.)

